# 4a-Methyl-2,3,4,4a-tetra­hydro-1*H*-carbazole-6-sulfonamide

**DOI:** 10.1107/S1600536812010239

**Published:** 2012-03-14

**Authors:** Abdulrahman O. Al-Youbi, Abdullah M. Asiri, Hassan M. Faidallah, Seik Weng Ng, Edward R. T. Tiekink

**Affiliations:** a Chemistry Department, Faculty of Science, King Abdulaziz University, PO Box 80203, Jeddah, Saudi Arabia; b The Center of Excellence for Advanced Materials Research, King Abdulaziz University, Jeddah, PO Box 80203, Saudi Arabia; c Department of Chemistry, University of Malaya, 50603 Kuala Lumpur, Malaysia

## Abstract

In the title mol­ecule, C_13_H_16_N_2_O_2_S, the nine non-H atoms comprising the indole residue are approximately coplanar (r.m.s. deviation = 0.031 Å). The partially saturated ring adopts a chair conformation. One amine H forms an inter­molecular N—H⋯O hydrogen bond to a sulfonamide O atom, while the other amine H form is connected to the indole N atom of an adjacent mol­ecule *via* an N—H⋯N hydrogen bond, resulting in a three-dimensional architecture.

## Related literature

For background to the biological applications of related sulfonamides, see: Al-Saadi *et al.* (2008 ▶). For related structures, see: Asiri *et al.* (2011 ▶, 2012 ▶).
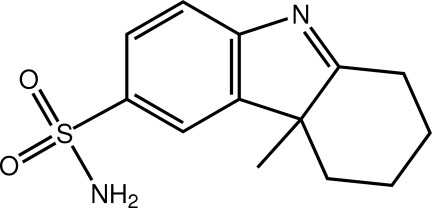


## Experimental

### 

#### Crystal data

C_13_H_16_N_2_O_2_S
*M* *_r_* = 264.34
Monoclinic, 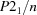

*a* = 9.3694 (5) Å
*b* = 10.4051 (5) Å
*c* = 13.5937 (8) Å
β = 103.516 (6)°
*V* = 1288.54 (12) Å^3^
*Z* = 4
Mo *K*α radiation
μ = 0.25 mm^−1^
*T* = 100 K
0.25 × 0.20 × 0.10 mm

#### Data collection

Agilent SuperNova Dual diffractometer with an Atlas detector
Absorption correction: multi-scan (*CrysAlis PRO*; Agilent, 2011 ▶) *T*_min_ = 0.941, *T*_max_ = 0.976
5426 measured reflections
2959 independent reflections
2364 reflections with *I* > 2σ(*I*)
*R*_int_ = 0.030

#### Refinement

*R*[*F* ^2^ > 2σ(*F* ^2^)] = 0.045
*wR*(*F* ^2^) = 0.121
*S* = 1.05
2959 reflections
171 parameters
H atoms treated by a mixture of independent and constrained refinement
Δρ_max_ = 0.79 e Å^−3^
Δρ_min_ = −0.48 e Å^−3^

### 

Data collection: *CrysAlis PRO* (Agilent, 2011 ▶); cell refinement: *CrysAlis PRO*; data reduction: *CrysAlis PRO*; program(s) used to solve structure: *SHELXS97* (Sheldrick, 2008 ▶); program(s) used to refine structure: *SHELXL97* (Sheldrick, 2008 ▶); molecular graphics: *ORTEP-3* (Farrugia, 1997 ▶) and *DIAMOND* (Brandenburg, 2006 ▶); software used to prepare material for publication: *publCIF* (Westrip, 2010 ▶).

## Supplementary Material

Crystal structure: contains datablock(s) global, I. DOI: 10.1107/S1600536812010239/xu5479sup1.cif

Structure factors: contains datablock(s) I. DOI: 10.1107/S1600536812010239/xu5479Isup2.hkl

Supplementary material file. DOI: 10.1107/S1600536812010239/xu5479Isup3.cml

Additional supplementary materials:  crystallographic information; 3D view; checkCIF report

## Figures and Tables

**Table 1:** Hydrogen-bond geometry (Å, °)

*D*—H⋯*A*	*D*—H	H⋯*A*	*D*⋯*A*	*D*—H⋯*A*
N2—H1⋯N1^i^	0.86 (3)	2.13 (3)	2.986 (3)	170 (2)
N2—H2⋯O1^ii^	0.86 (3)	2.20 (3)	3.039 (2)	164 (2)

Symmetry codes: (i) 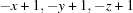; (ii) 
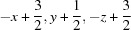
.

**Fig. 1.**
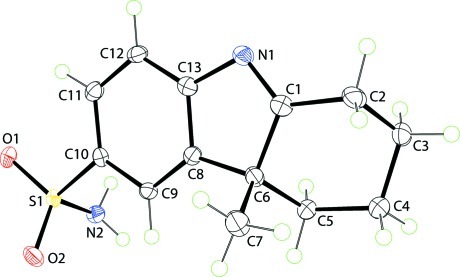
The molecular structure of (I) showing the atom-labelling scheme and displacement ellipsoids at the 50% probability level.

**Fig. 2.**
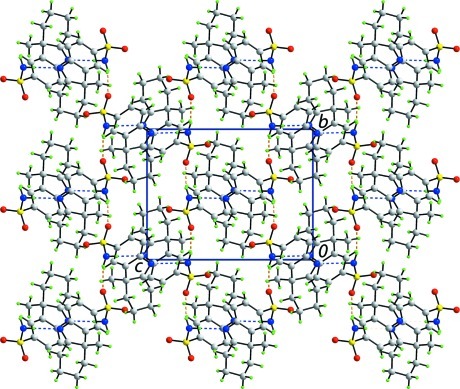
A view in projection down the a axis of the unit-cell contents of (I). The N—H···O and N—H···N interactions are shown as orange and blue dashed lines, respectively.

## supplementary crystallographic information

### Comment

Sulphonamides related to the title compound, 4*b*-methyl-5,6,7,8-tetrahydro-4*H*-carbazole-3-sulfonic acid amide (I), are known to possess biological activity (Al-Saadi *et al.*, 2008). In continuation of structural studies of these systems (Asiri *et al.*, 2011; Asiri *et al.*, 2012), the crystal and molecular structure of (I) is reported herein.

In (I), Fig. 1, the partially saturated ring adopts the conformation of a chair. The nine non-carbon atoms of the indole residue are co-planar, having a r.m.s. deviation of 0.031 Å. With reference to this plane, the C1—C6 ring and the amino group lie to one side, with the methyl group and one sulphonamide-O atom being orientated to the other.

Strong hydrogen bonding interactions dominate the crystal packing. Thus, one amino-H forms a hydrogen bond to the sulphonamide-O1 atom while the others forms a hydrogen bond to the indole-N atom, Table 1. The result is a three-dimensional architecture, Fig. 2.

### Experimental

1-Methylcyclohexanone (1.1 g, 10 mmol) in ethanol was refluxed with *p*-sulfamylphenylhydrazine (2.2 g, 10 mmol) for 1 h. The reaction mixture was cooled and the precipitated solid product was collected by filtration, washed with ethanol, dried and recrystallized from ethanol. Yield: 78%.

### Refinement

Carbon-bound H-atoms were placed in calculated positions [C—H = 0.95 to 0.99 Å, *U*_iso_(H) = 1.2*U*_eq_(C)] and were included in the refinement in the riding model approximation. The amino H-atoms were located in a difference Fourier map, and were refined with a distance restraint of N—H = 0.88±0.01 Å; their *U*_iso_ values were refined.

### Crystal data

**Table:**

C_13_H_16_N_2_O_2_S	*F*(000) = 560
*M**_r_* = 264.34	*D*_x_ = 1.363 Mg m^−^^3^
Monoclinic, *P*2_1_/*n*	Melting point = 513–514 K
Hall symbol: -P 2yn	Mo *K*α radiation, λ = 0.71073 Å
*a* = 9.3694 (5) Å	Cell parameters from 2450 reflections
*b* = 10.4051 (5) Å	θ = 2.4–27.5°
*c* = 13.5937 (8) Å	µ = 0.25 mm^−^^1^
β = 103.516 (6)°	*T* = 100 K
*V* = 1288.54 (12) Å^3^	Prism, light-brown
*Z* = 4	0.25 × 0.20 × 0.10 mm

### Data collection

**Table:**

Agilent SuperNova Dual diffractometer with an Atlas detector	2959 independent reflections
Radiation source: SuperNova (Mo) X-ray Source	2364 reflections with *I* > 2σ(*I*)
Mirror monochromator	*R*_int_ = 0.030
Detector resolution: 10.4041 pixels mm^-1^	θ_max_ = 27.6°, θ_min_ = 2.4°
ω scan	*h* = −8→12
Absorption correction: multi-scan (*CrysAlis PRO*; Agilent, 2011)	*k* = −10→13
*T*_min_ = 0.941, *T*_max_ = 0.976	*l* = −17→17
5426 measured reflections	

### Refinement

**Table:**

Refinement on *F*^2^	Primary atom site location: structure-invariant direct methods
Least-squares matrix: full	Secondary atom site location: difference Fourier map
*R*[*F*^2^ > 2σ(*F*^2^)] = 0.045	Hydrogen site location: inferred from neighbouring sites
*wR*(*F*^2^) = 0.121	H atoms treated by a mixture of independent and constrained refinement
*S* = 1.05	*w* = 1/[σ^2^(*F*_o_^2^) + (0.0503*P*)^2^ + 0.7098*P*] where *P* = (*F*_o_^2^ + 2*F*_c_^2^)/3
2959 reflections	(Δ/σ)_max_ = 0.001
171 parameters	Δρ_max_ = 0.79 e Å^−^^3^
0 restraints	Δρ_min_ = −0.48 e Å^−^^3^

### Fractional atomic coordinates and isotropic or equivalent isotropic displacement parameters (Å^2^)

**Table:**

	*x*	*y*	*z*	*U*_iso_*/*U*_eq_	
S1	0.72173 (5)	0.36862 (5)	0.76427 (4)	0.01546 (15)	
O1	0.76980 (15)	0.24573 (13)	0.73458 (11)	0.0199 (3)	
N2	0.8396 (2)	0.47419 (18)	0.74549 (15)	0.0183 (4)	
N1	0.17508 (18)	0.53036 (16)	0.47626 (13)	0.0174 (4)	
O2	0.70440 (16)	0.38463 (14)	0.86604 (11)	0.0226 (4)	
C1	0.1424 (2)	0.63100 (19)	0.52284 (16)	0.0170 (4)	
C2	0.0292 (2)	0.7272 (2)	0.47752 (16)	0.0228 (5)	
H2A	−0.0452	0.7341	0.5184	0.027*	
H2B	−0.0209	0.7011	0.4080	0.027*	
C3	0.1071 (3)	0.8567 (2)	0.47558 (17)	0.0230 (5)	
H3A	0.1717	0.8516	0.4276	0.028*	
H3B	0.0329	0.9244	0.4513	0.028*	
C4	0.1988 (2)	0.8935 (2)	0.58062 (16)	0.0223 (5)	
H4A	0.2484	0.9766	0.5762	0.027*	
H4B	0.1332	0.9045	0.6275	0.027*	
C5	0.3141 (2)	0.79111 (19)	0.62255 (16)	0.0186 (4)	
H5A	0.3676	0.8158	0.6917	0.022*	
H5B	0.3859	0.7867	0.5796	0.022*	
C6	0.2435 (2)	0.65700 (19)	0.62602 (15)	0.0164 (4)	
C7	0.1608 (2)	0.6485 (2)	0.71120 (17)	0.0235 (5)	
H7A	0.1174	0.5628	0.7112	0.035*	
H7B	0.0828	0.7134	0.7001	0.035*	
H7C	0.2293	0.6640	0.7765	0.035*	
C8	0.3498 (2)	0.54740 (18)	0.62982 (15)	0.0143 (4)	
C9	0.4757 (2)	0.51408 (18)	0.70010 (15)	0.0147 (4)	
H9	0.5094	0.5633	0.7599	0.018*	
C10	0.5525 (2)	0.40494 (18)	0.68035 (15)	0.0143 (4)	
C11	0.5027 (2)	0.32996 (19)	0.59393 (15)	0.0176 (4)	
H11	0.5554	0.2552	0.5834	0.021*	
C12	0.3755 (2)	0.36502 (18)	0.52309 (16)	0.0168 (4)	
H12	0.3402	0.3151	0.4639	0.020*	
C13	0.3022 (2)	0.47451 (19)	0.54154 (15)	0.0157 (4)	
H1	0.847 (3)	0.474 (2)	0.683 (2)	0.032 (7)*	
H2	0.816 (3)	0.549 (3)	0.764 (2)	0.030 (7)*	

### Atomic displacement parameters (Å^2^)

**Table:**

	*U*^11^	*U*^22^	*U*^33^	*U*^12^	*U*^13^	*U*^23^
S1	0.0175 (3)	0.0139 (3)	0.0143 (3)	0.00338 (19)	0.00238 (19)	0.00179 (18)
O1	0.0226 (8)	0.0133 (7)	0.0235 (8)	0.0049 (6)	0.0049 (6)	0.0014 (6)
N2	0.0192 (9)	0.0167 (9)	0.0180 (10)	−0.0003 (7)	0.0025 (7)	−0.0014 (7)
N1	0.0159 (8)	0.0189 (8)	0.0160 (9)	0.0011 (7)	0.0009 (7)	−0.0010 (7)
O2	0.0280 (8)	0.0262 (8)	0.0137 (8)	0.0064 (7)	0.0048 (6)	0.0038 (6)
C1	0.0135 (9)	0.0201 (10)	0.0175 (10)	−0.0014 (8)	0.0037 (8)	−0.0002 (8)
C2	0.0194 (10)	0.0282 (11)	0.0179 (11)	0.0073 (9)	−0.0014 (8)	−0.0030 (9)
C3	0.0285 (12)	0.0216 (11)	0.0185 (11)	0.0103 (9)	0.0043 (9)	0.0015 (8)
C4	0.0269 (11)	0.0200 (10)	0.0196 (11)	0.0057 (9)	0.0050 (9)	−0.0009 (9)
C5	0.0226 (11)	0.0159 (10)	0.0172 (10)	0.0030 (9)	0.0046 (8)	−0.0006 (8)
C6	0.0176 (10)	0.0180 (10)	0.0136 (10)	0.0039 (8)	0.0038 (8)	0.0016 (8)
C7	0.0232 (11)	0.0281 (11)	0.0208 (11)	0.0069 (9)	0.0084 (9)	0.0025 (9)
C8	0.0160 (9)	0.0138 (9)	0.0145 (10)	−0.0001 (8)	0.0061 (8)	0.0006 (7)
C9	0.0178 (10)	0.0148 (9)	0.0120 (9)	−0.0006 (8)	0.0043 (8)	−0.0007 (7)
C10	0.0142 (9)	0.0146 (9)	0.0144 (10)	0.0012 (8)	0.0040 (7)	0.0035 (8)
C11	0.0208 (10)	0.0143 (9)	0.0191 (11)	0.0007 (8)	0.0078 (8)	−0.0011 (8)
C12	0.0198 (10)	0.0153 (10)	0.0155 (10)	−0.0036 (8)	0.0043 (8)	−0.0032 (8)
C13	0.0150 (9)	0.0179 (10)	0.0145 (10)	−0.0024 (8)	0.0038 (8)	0.0005 (8)

### Geometric parameters (Å, º)

**Table:**

S1—O2	1.4398 (15)	C4—H4B	0.9900
S1—O1	1.4442 (14)	C5—C6	1.549 (3)
S1—N2	1.6197 (19)	C5—H5A	0.9900
S1—C10	1.764 (2)	C5—H5B	0.9900
N2—H1	0.86 (3)	C6—C8	1.507 (3)
N2—H2	0.86 (3)	C6—C7	1.539 (3)
N1—C1	1.297 (3)	C7—H7A	0.9800
N1—C13	1.432 (3)	C7—H7B	0.9800
C1—C2	1.484 (3)	C7—H7C	0.9800
C1—C6	1.522 (3)	C8—C9	1.377 (3)
C2—C3	1.536 (3)	C8—C13	1.401 (3)
C2—H2A	0.9900	C9—C10	1.403 (3)
C2—H2B	0.9900	C9—H9	0.9500
C3—C4	1.533 (3)	C10—C11	1.396 (3)
C3—H3A	0.9900	C11—C12	1.395 (3)
C3—H3B	0.9900	C11—H11	0.9500
C4—C5	1.529 (3)	C12—C13	1.383 (3)
C4—H4A	0.9900	C12—H12	0.9500
			
O2—S1—O1	118.92 (9)	C4—C5—H5B	109.3
O2—S1—N2	107.89 (10)	C6—C5—H5B	109.3
O1—S1—N2	106.72 (10)	H5A—C5—H5B	107.9
O2—S1—C10	108.08 (9)	C8—C6—C1	99.28 (16)
O1—S1—C10	107.50 (9)	C8—C6—C7	112.08 (16)
N2—S1—C10	107.21 (9)	C1—C6—C7	111.62 (17)
S1—N2—H1	111.6 (17)	C8—C6—C5	113.55 (16)
S1—N2—H2	109.5 (17)	C1—C6—C5	108.06 (16)
H1—N2—H2	112 (2)	C7—C6—C5	111.58 (17)
C1—N1—C13	106.40 (17)	C6—C7—H7A	109.5
N1—C1—C2	124.71 (19)	C6—C7—H7B	109.5
N1—C1—C6	115.23 (18)	H7A—C7—H7B	109.5
C2—C1—C6	119.57 (18)	C6—C7—H7C	109.5
C1—C2—C3	107.61 (17)	H7A—C7—H7C	109.5
C1—C2—H2A	110.2	H7B—C7—H7C	109.5
C3—C2—H2A	110.2	C9—C8—C13	120.48 (18)
C1—C2—H2B	110.2	C9—C8—C6	131.77 (18)
C3—C2—H2B	110.2	C13—C8—C6	107.74 (17)
H2A—C2—H2B	108.5	C8—C9—C10	117.72 (18)
C4—C3—C2	111.61 (18)	C8—C9—H9	121.1
C4—C3—H3A	109.3	C10—C9—H9	121.1
C2—C3—H3A	109.3	C11—C10—C9	121.85 (18)
C4—C3—H3B	109.3	C11—C10—S1	119.82 (15)
C2—C3—H3B	109.3	C9—C10—S1	118.23 (15)
H3A—C3—H3B	108.0	C12—C11—C10	119.89 (18)
C5—C4—C3	111.52 (17)	C12—C11—H11	120.1
C5—C4—H4A	109.3	C10—C11—H11	120.1
C3—C4—H4A	109.3	C13—C12—C11	118.07 (19)
C5—C4—H4B	109.3	C13—C12—H12	121.0
C3—C4—H4B	109.3	C11—C12—H12	121.0
H4A—C4—H4B	108.0	C12—C13—C8	121.94 (18)
C4—C5—C6	111.71 (17)	C12—C13—N1	126.75 (18)
C4—C5—H5A	109.3	C8—C13—N1	111.29 (17)
C6—C5—H5A	109.3		
			
C13—N1—C1—C2	172.04 (19)	C13—C8—C9—C10	−0.6 (3)
C13—N1—C1—C6	0.1 (2)	C6—C8—C9—C10	−179.19 (19)
N1—C1—C2—C3	−117.4 (2)	C8—C9—C10—C11	−1.4 (3)
C6—C1—C2—C3	54.2 (2)	C8—C9—C10—S1	174.85 (15)
C1—C2—C3—C4	−54.0 (2)	O2—S1—C10—C11	−139.60 (16)
C2—C3—C4—C5	58.2 (2)	O1—S1—C10—C11	−10.09 (19)
C3—C4—C5—C6	−56.1 (2)	N2—S1—C10—C11	104.33 (17)
N1—C1—C6—C8	1.3 (2)	O2—S1—C10—C9	44.03 (18)
C2—C1—C6—C8	−171.03 (18)	O1—S1—C10—C9	173.54 (15)
N1—C1—C6—C7	−117.0 (2)	N2—S1—C10—C9	−72.03 (17)
C2—C1—C6—C7	70.6 (2)	C9—C10—C11—C12	1.8 (3)
N1—C1—C6—C5	119.95 (19)	S1—C10—C11—C12	−174.46 (15)
C2—C1—C6—C5	−52.4 (2)	C10—C11—C12—C13	0.0 (3)
C4—C5—C6—C8	159.10 (17)	C11—C12—C13—C8	−2.0 (3)
C4—C5—C6—C1	50.0 (2)	C11—C12—C13—N1	176.45 (19)
C4—C5—C6—C7	−73.1 (2)	C9—C8—C13—C12	2.3 (3)
C1—C6—C8—C9	176.5 (2)	C6—C8—C13—C12	−178.76 (18)
C7—C6—C8—C9	−65.5 (3)	C9—C8—C13—N1	−176.32 (18)
C5—C6—C8—C9	62.1 (3)	C6—C8—C13—N1	2.6 (2)
C1—C6—C8—C13	−2.2 (2)	C1—N1—C13—C12	179.7 (2)
C7—C6—C8—C13	115.73 (19)	C1—N1—C13—C8	−1.7 (2)
C5—C6—C8—C13	−116.70 (19)		

### Hydrogen-bond geometry (Å, º)

**Table:**

*D*—H···*A*	*D*—H	H···*A*	*D*···*A*	*D*—H···*A*
N2—H1···N1^i^	0.86 (3)	2.13 (3)	2.986 (3)	170 (2)
N2—H2···O1^ii^	0.86 (3)	2.20 (3)	3.039 (2)	164 (2)

Symmetry codes: (i) −*x*+1, −*y*+1, −*z*+1; (ii) −*x*+3/2, *y*+1/2, −*z*+3/2.
